# Supplementary material for: The perceived impact of isoniazid resistance on outcome of first-line rifampicin-throughout regimens is largely due to missed rifampicin resistance
Source: PLoS One. 2020 May 18;15(5):e0233500. doi: 10.1371/journal.pone.0233500 (PMC7233532; doi:10.1371/journal.pone.0233500)
Supplement: Attachment — Submitted filename: PONE-D-20-07659.edited.docx [file pone.0233500.s001.docx]

**Comments to the authors**

The study by Armand Van Deun et al. studied a cohort of 7291 patients from Bangladesh for the prediction of various tuberculosis resistance, mainly the involvement of initial isoniazid resistance, rifampicin-resistant, as well as multi-drug resistance in the success of various treatment regimens. The authors mentioned the success of throughout rifampicin resistance in the treatment of isoniazid-resistant TB as well as a failure of this regimen in the treatment of missed rifampicin and acquired rifampicin-resistant TB cases. Importantly, the authors mentioned that failure of other regimens, including throughout rifampicin treatment in the initially missed cases of rifampicin-resistant or acquired resistance to rifampicin, is mainly due to the detection limit in the determination of rifampicin resistance. It is evidenced from the published literature that Rifampicin resistance is commonly used as a predictor of multi-drug resistance in *M. tb*, also confirmed by the analysis of this study. The study is well conducted; data is well organized and presented. The manuscript is well written and understandable except the abstract that can be written in a more straightforward way to be useful for the wider audience

**Abstract:**

The abstract written is quite complicated. It is hard to understand. Authors are requested to write an abstract that can easily be understood.
