# Supplementary material for: The perceived impact of isoniazid resistance on outcome of first-line rifampicin-throughout regimens is largely due to missed rifampicin resistance
Source: PLoS One. 2020 May 18;15(5):e0233500. doi: 10.1371/journal.pone.0233500 (PMC7233532; doi:10.1371/journal.pone.0233500)
Supplement: Attachment — Submitted filename: Response to reviewers.docx [file pone.0233500.s002.docx]

PONE-D-20-07659
The perceived impact of isoniazid resistance on outcome of first-line rifampicin-throughout regimens is largely due to missed rifampicin resistance

**Response to the reviewers**

Reviewer #1: Comments to the authors
The study by Armand Van Deun et al. studied a cohort of 7291 patients from Bangladesh for the prediction of various tuberculosis resistance, mainly the involvement of initial isoniazid resistance, rifampicin-resistant, as well as multi-drug resistance in the success of various treatment regimens. The authors mentioned the success of throughout rifampicin resistance in the treatment of isoniazid-resistant TB as well as a failure of this regimen in the treatment of missed rifampicin and acquired rifampicin-resistant TB cases. Importantly, the authors mentioned that failure of other regimens, including throughout rifampicin treatment in the initially missed cases of rifampicin-resistant or acquired resistance to rifampicin, is mainly due to the detection limit in the determination of rifampicin resistance. It is evidenced from the published literature that Rifampicin resistance is commonly used as a predictor of multi-drug resistance in M. tb, also confirmed by the analysis of this study. The study is well conducted; data is well organized and presented. The manuscript is well written and understandable except the abstract that can be written in a more straightforward way to be useful for the wider audience
Abstract:
The abstract written is quite complicated. It is hard to understand. Authors are requested to write an abstract that can easily be understood.

*Thank you so much for this important remark. We have revised the abstract extensively so as to make it easily understandable.*

Reviewer #2: Comments:
Overall the manuscript is broadly written about the treatment categories related to active TB. Data proved the treatment utility and recommendations in present scenario for TB programme successfully progresses worldwide. Following are some suggestions which can make the manuscript stronger and beneficial for the readers:

Abstract:
• Abbreviation of rifampicin resistance (Rr) should come upon first appearance in the text.

*We are sorry for this lapse, it has been corrected.*

• Conclusive statement should be modified to give good interpretation to the reader.

*The abstract has been revised entirely, including the conclusion, as advised by Reviewer 1. Moreover, since we stress the danger of acquired fluoroquinolone resistance as more important than the negative impact of isoniazid resistance, we thought this needed some more explanation. We have added a few sentences to explain why acquired resistance could come up quite readily and frequently in the discussion about the strengthened regimen.*

Introduction:
• Abbreviation must require for rifampicin (RMP) upon first appearance in the text (Line no 60).

*We are sorry for this lapse, it has been corrected.*

Method: It observed that author/s ware collected the data very precisely. However, required some information to improve manuscript like:
• Author should point out the type (pulmonary or extra-pulmonary) of tuberculosis among patient requited for this study as well as house hold contact, how developed active TB.

*Thank you. All patients were pulmonary cases, and this is now specified in the manuscript. Unfortunately contacts and secondary cases that developed from them were as a rule not registered in the computer database, although they were listed on the original paper treatment cards that we couldn't analyse. This may not be a serious deficiency because outside the systematic population surveys, in fact only contacts of MDR cases were tested for baseline drug resistance. This contact status has been recorded in the database and we have now added their number included in the study in the text (methods as well as results).*

• In patient history, alcoholism and smoker status of patient as well as house hold contact should be noted if possible. Because these habits are strongly associated with TB disease and attributed to high risk of TB infection which leads to active TB or recurrence.

*We are sorry, but much of this information is not available because it all started as an observational study, with close to 300,000 cases individually entered into the original patient database, focusing on the variables considered to be most important for analyses of diagnostic and treatment parameters only, not risk factors for the disease or recurrence. Smoking, occupation and household contact were thus not part of the computerised database variables and therefore unavailable for analysis. However, we can confidently state that alcoholism exists in this largely Muslim country only among tribal non-Muslim minorities, very few of whom are included in our dataset. While smoking is very common among males.*

Results:
• Authors should provide the data of TB-HIV co-infected patients. Because, they may have negative in sputum smear microscopy and required initial TB treatment.

*We apologise again, because HIV status in Bangladeshi TB patients has been determined only in the context of ad hoc studies. That consistently showed a very low prevalence of the order of 1 per 1000 patients, explaining that routine testing of all patients was not considered cost-effective by the TB control programme authorities. We have added this fact, and that HIV thus cannot have influenced our findings.*
